# Vitality and Immunity

**Published:** 1903-04

**Authors:** Carl Schulin

**Affiliations:** Billings, Mont.


### VITALITY AND IMMUNITY.

By Carl Schulin, M.D.,
BILLINGS, MONT.

(Concluded from page 156.)

Like a true savant, Goetsch kept silent for ten years and published his results only after the last doubt of success had been removed. His paper appeared in the *Deutsche Medicinische Wochenschrift*, 1901, No. 25. It is followed by a few words of Koch, who says that quite a number of observers found that they invariably obtained good results from tuberculin in cases not complicated with suppuration, not too far advanced, and free from fever. They all agreed that the dose should be very small.

The tubercle bacillus has many features in common with the anthrax bacilllus. Both are aërobic, and the products of the metabolism of both are non-poisonous. Both injure their hosts in the same way, namely, with their affinity to oxygen. When poisons are formed they originate from decomposition of tissues. They are products of necrobiosis, not excretions of bacteria. The anthrax bacillus lives principally in the blood and feeds on the oxygen which the body receives through the lungs. The tubercle bacillus feeds, facultative-anaërobically, on the above-mentioned fifth of oxygen, which apparently is furnished, in and around nuclei, by processes of reduction. The tubercle bacillus always stays in and around nuclei. It interferes with the production of spermin and lives on the very heart of vitality.

When we compare the course of Koch's artificially produced guinea-pig tuberculosis with that of human tuberculosis, we see a striking difference. Koch's guinea-pig tuberculosis is an absolutely fatal disease. Its initial symptoms resemble those of syphilis, with the difference that the tuberculous chancre never heals. Human tuberculosis has only one feature in common with syphilis, that of producing indolent buboes, but it has no chancre. The bacilli, in a case of human tuberculosis, do not enter into the body suddenly, *en masse*, and, as a rule, not through an open wound. They sneak in singly, here and there, and leave no trace behind at the place of their entrance. Quietly they take possession of lymphatic glands or some other parts and make themselves at home.

The human tubercle bacilli alone seem unable to kill men. They produce locally some necrobiosis and remain in the cheesy matter thus produced in a dormant condition. They seem to be unable to resist the slow, but persistent influence of actinic rays which may reach them, and of the now and then appearing leucocytes. They succumb finally and spontaneous cure results. To accomplish the destructive work for which they are so much dreaded, they apparently need some assistance. Obviously they receive this from different kinds of cocci.

The reader is familiar with the way in which cocci enter into the body. As a rule, they are arrested and destroyed in the nearest lymphatic glands, except when they are so virulent as to produce lymphangitis, lymphadenitis, and pyæmia. Sometimes they pass the lymphatic glands and produce inflammation of inner organs. They favor the serous membranes. Pleurisy and some forms of articular rheumatism have been found to be caused by cocci.

When the lymphatic glands are infected with tubercle bacilli, the cocci seem to pass more easily through them and to carry the bacilli with them into the circulation. In the union with the cocci we have to look for the answer to the above-proposed question, why the tubercle bacilli sometimes so suddenly become virulent and spread all over the body.

The mixed infection is the cause of the destruction of the body. It produces the tuberculous ulcerations in the bowels and the bronchial tubes. They are secondary, not primary, lesions. The union of the tubercle bacilli with the cocci is manifested by fever. The tubercle bacilli alone cannot produce fever. Fever is a coccus symptom.

Therefore, it is necessary to recognize and cure tuberculosis before the tubercle bacilli have entered into partnership with the cocci. Both can be done with the same means—with Koch's old tuberculin. In the treatment the actinic rays of light should be combined with the tuberculin.

However deplorable it may be that through human error so great a discovery as Koch's tuberculin has been denied proper recognition for more than a decade, over the gloomy past we must not forget the bright future. Through the parting clouds of disappointments and mistakes, to-day we see our way clear to the realization of one of the greatest objects in preventive medicine, namely, the extermination of tuberculosis. Together with Jenner and Lister, Robert Koch will forever be named as one of the greatest benefactors of the human race!

In our enthusiasm, however, we must not forget the enormous difficulties which we have yet to overcome. It will be slow work to regain the confidence of the disappointed medical fraternity, as well as the public generally. Besides we shall find great difficulties in the early recognition of tuberculosis in young children of apparently good health. A hypodermic test-injection in a case like the above-mentioned delicate little girl of good family will meet with the combined resistance of the mother and the young lady. Here Poehl's coefficient comes in handily. Tuberculosis and a high coefficient can hardly exist together. If the coefficient is low we have to look for enlarged lymphatic glands, tonsils, and follicles, and to call the attention of the parents to the dangers threatening therefrom.

One of the most charming subjects in pathology is the *modus operandi* of tuberculin. Here the first question which confronts us is, Why do the tubercle bacilli not of themselves immunize their host, if it is possible with their products? The possibility that in some cases of spontaneous cure the bacilli might have done this, cannot be denied, but, as a rule, they certainly do not do it. The writer looks for the reason of this in their small number and their comparatively inactive condition. Only after the tubercle bacilli have allied themselves with the cocci does their number increase rapidly. The cocci, however, seem to interfere with the immunization against the tubercle bacilli. This appears from the fact that tuberculin takes effect only before the tubercle bacilli have united with the cocci, or after the cocci have been subdued, but not while both co-operate in full strength.

The next question is, Why does tuberculin produce fever only in a body infected with tubercle bacilli and not in a healthy one? For a possible solution of this puzzle we must go back a little.

As stated above, we are fairly well informed as to the different stages of the retrogressive metamorphosis of albumin through processes of oxidation and hydration. Besides this, however, there is another retrogressive metamorphosis of albumin, namely, through processes of reduction. While the former terminates with compounds rich in oxygen—*e. g.*, urea, carbonic acid, and water—the latter terminates with compounds free from oxygen—*e. g.*, ammonia, hydrocarbons, sulphuretted hydrogen, etc. There is not much known of the intermediary stages of the retrograde reduction metamorphosis. The writer believes that the ptomaines which Poehl puts together with the leukomaines, belong on the reduction side, while the leukomaines belong on the oxidation side.

The oxidation metamorphosis takes place in the protoplasma of living animal and vegetable cells. It is a symptom of life. The reduction metamorphosis takes place in dead albumin under the influence of saprophytic germs. It seems that the products of oxidation metamorphosis all have positive chemotactic force, while those of reduction metamorphosis have negative chemotactic force.

As said before, the first changes from retrogressive metamorphosis are of a physical and not as yet of a chemical nature. Some of the thus changed albumins are very poisonous, and are, therefore, called toxalbumins. No one, however, seems to have noticed that there ought to be two kinds of such physically changed albumin, one leaning toward oxidation and one toward reduction. The writer believes that only those leaning toward reduction are poisonous, while the others are harmless.

In plants a third, similar, metamorphosis occurs, but a progressive one. Plants form albumin, apparently commencing with plain nitrogen, and add other elements and molecules to it, with an upbuilding tendency. Nothing seems to be known of the intermediary stages in the formation of vegetable albumin. We only see that processes of oxidation and reduction work together in a very complicated way. We see that growing plants absorb carbonic acid, reduce it to carbon oxide, excrete oxygen and implant the former into a developing molecule of albumin or carbon hydrate. Apparently this is done by the action of some ferment which originates in the nuclei of certain cells.

The contrast between processes of oxidation and processes of reduction seems to be the essential principle of life. Its elimination means death. This contrast shows itself in the different functions of the different parts of the cell. The protoplasma does its work through processes of retrogressive oxidation metamorphosis, while the nucleus attends to repairing and upbuilding through processes of progressive reduction metamorphosis. The reader will understand how easily, through products of commencing retrogressive reduction metamorphosis, the contrast between protoplasma and nucleus may be eliminated and death caused.

The nucleus, in the nucleo-albumin, contains an albuminous substance which has a much more complicated structure than any of its kin in the protoplasma. And the nucleo-albumin contains, as its molecular nucleus, the spermin, a ferment which has the power of promoting oxidation at one pole and reduction at another. Thus spermin is the chemical nucleus of vitality and the real weapon with which the body protects itself against infection.

Tuberculin, in the writer's opinion, is an oxidation albumin, while the cheesy matter in which the tubercle bacilli are imbedded, contains reduction albumin. Tuberculin is, therefore, non-poisonous, and has positive chemotactic force, but it exists inside of the germs and surrounded by cheesy matter with negative chemotactic force, which keeps the leucocytes away. If this matter is soaked with tuberculin from the outside the leucocytes will be attracted. Inflammation and fever from reabsorption of inflammatory products will be the result. Healthy leucocytes can now destroy the tubercle bacilli. Leucocytes which are weakened by a fight with cocci will not be able to do it. This explains satisfactorily why tuberculin makes no symptoms at all in a healthy body and why it cannot exert its curative influence in cases of mixed infection.

The nature of the immunity which can be produced with tuberculin against the tubercle bacillus is not fully understood. It resembles the immunity against anthrax. There is no tubercle antitoxin; in the same way there is no anthrax antitoxin. The immunity is based on questions of oxidation and chemotaxis, as it is in the case of anthrax. The reader will notice that the abovementioned conditions by which the immunity against anthrax is destroyed, namely, exhaustion from overwork, inanition, refrigeration, etc., all tend to make spermin inert. On the other side, tuberculin, as an oxidation albumin with positive chemotactic force, will tend to increase the number of the leucocytes in the blood and thus stimulate the production of spermin.

In immunity a fundamental distinction should be made between the general power of resistance of the body against infection of any kind and the immunities against special diseases. The former, which is identical with the old-time "vitality," is furnished by certain protective arrangements. While looking at it from a standpoint entirely different from that of Brown-Séquard, Buchner also recognized that the underlying cause of this power of resistance has a chemical nature. He assumed that it is based on the presence, in the blood serum of certain protective substances which he named "alexins." Buchner also recognized that these substances are produced by the leucocytes, but he did not succeed in isolating them.

Kossel found that a less than one-half per cent. solution of nucleo-albumin which he obtained from calves' leucocytes, has strong germicidal action. Vaughan and McClintock found that nuclein kills germs. Finally, Poehl discovered in spermin the source of the germicidal action of the tissues. He traced its origin to the nuclei, especially of leucocytes, and located exactly the spot where it stands in the nucleo-albumin between the proteins and other compounds. Protoplasma apparently produces no spermin; only the nuclei do.

Special immunities originate during the diseases concerned. They can also be produced artificially by attenuated cultures of germs and with the toxins alone. Their characteristic feature is the presence, in the blood serum, of newly-formed substances which neutralize the effects of the germs (active immunity). Authors generally distinguish between two kinds of active immunity, namely: (1) The resistance against the toxins, and (2) the power of destroying the germs.

After recovery from diseases in the course of which very active poisons are formed, the blood contains substances which are antagonistic to them. These are the antitoxins of which we treated before. The writer gave his reasons why he, with Ehrlich, believes they are newly-formed products of the diseased body, not changed toxins. The toxins are generally taken for products of the germs. The writer, however, believes that mostly all of those toxins which really deserve that name, namely, the poisonous ones, originate also in the diseased body. They are not, like the antitoxins, produced actively by the metabolism, but they originate passively in tissues killed and decomposed under the influence of germs. They are products of retrogressive reduction metamorphosis and, therefore, poisonous and with negative chemotactic force. The wrong idea that they were products of the bacteria was probably caused by their also being found in pure cultures of germs; but why should they not originate here in the nutrient?

The word "toxin" is used too generally in the same sense as "products" of bacteria. Everybody speaks of tuberculin as a toxin, although it has no toxic qualities at all. Any toxalbumin present should be considered as a dangerous impurity.

Ehrlich, recently, in the *Deutsche medicinische Wochenschrift*, 1901,

Nos. 50 to 52, published a synopsis of the latest facts and theories concerning the protective substances of the blood. The narrow limits of this paper do not permit of entering deeper into his arguments, which are rather obscured by a complicated terminology. Suffice it, however, to say that he believes the toxins are not destroyed in the body, but arrested by certain molecules of the protoplasma which he calls side-chains (Seitenketten). Under this impulse other side-chains are formed, break loose, and float around in the blood as antitoxins.

Ehrlich is doubtless on the right track, but he ignores the investigations of Poehl about spermin, and those of Gautier, Kossel and others about albuminous bodies, especially nucleo-albumin. Otherwise he would have seen that protoplasma does not contain the material for the formation of "side-chains," but that the nuclei do!

An interesting fact was discovered by Pfeiffer and Marx, and corroborated by Wassermann, with regard to cholera, and also by Deutsch with regard to typhoid fever. During the process of immunization they found a stage in which the blood does not yet contain the protective bodies, but they exist already in the hæmopoetic organs, spleen, and bone-marrow.

Wassermann demonstrated that the spinal cord and brains arrest tetanus toxin. He showed that mixtures of tetanus toxin and emulsion of spinal cord are non-toxic. The spinal cords of animals which have died from tetanus contain antitoxin enough to protect other animals against the disease. He found that also in the test-tube a solution of tetanus toxin can be made non-poisonous by the addition of rubbed-up guinea-pig brain matter.

Still the toxin thus arrested is not destroyed. It is only chemically bound by the antitoxin, like a metal in a salt, and may reappear under certain conditions. Therefore Ehrlich uses the name of side-chains. In the antitoxin the reader will easily see the spermin!

How different the ways may be in which immunity can be obtained, appears from an interesting observation made by Ransom. He found that red blood corpuscles attract solanin with the cholesterin they contain and succumb afterward to its effects. Certain sera protect the blood against solanin poisoning, and they do this also with the cholesterin they contain.

Immune sera often contain, besides the antitoxins, some compounds which destroy bacteria under symptoms of swelling, paralysis, granular degeneration, and finally dissolution of their bodies. Fraenkel proposed for these substances the name of "lysogenic material." Other substances in immune sera cause, besides swelling and paralysis, a peculiar clogging together of the bacteria. These substances are called agglutinins. There is some discussion about the relation between the lysogenic material, the agglutinins, and the alexins. The solution will probably be found when the spermni gets the proper recognition and when we shall know more about the first changes in the structure of albumin during the two kinds of retrogressive metamorphosis.

The contents of this paper may be summarized as follows:

1. There is little stability of type in bacteria. They are changeable and adapt themselves in the course of time to new surroundings.

2. Pathogenic germs are descendants of saprophytic germs. Some of these germs, being transferred from man to man only, adapted themselves to their host so they cannot live elsewhere.

3. Cocci injure us with their positive chemotactic force, causing inflammation and fever, while the bacilli act with their negative chemotactic force and the poisonous products of retrogressive reduction metamorphosis, causing necrobiosis, gangrene, and death from poisoning of nervous centres.

4. The body protects itself against infection with spermin. The production, as well as the work of spermin, are under the influence of the actinic rays of light. Spermin is the nucleus of the nucleo-albumin as well as of the antitoxins.

5. Spermin does what our medical forefathers instinctively felt and, as they could not comprehend it, expressed with the word "vitality." Vitality is a time-honored word which should not be dropped, but should be honored the more the better we learn how to understand it. Preventive medicine is identical with preservation of vitality.

---

Treatment of Eczema in the Dog with Artificial Serum. Pécus applied this treatment in the dog. It had previously been used by Dr. Tommasoli with success in man. It consists of a subcutaneous injection of normal saline solution, and was employed in two cases. One dog, six years old, had in vain been treated in every manner for chronic eczema. A radical cure was obtained by injecting the saline solution, 7:1000, subcutaneously. The skin became supple, the coat rapidly grew out, and for three years there has been no evidence of the disease. In the second case the result was the same.—*Rev. Vet.*

Treatment of Warts. If a wart returns after excision, Pécus recommends the following ointment: Arsenious acid, powdered cantharides, each 1 part, turpentine 2 parts, oil and wax each 5 parts.

Two applications at intervals of a few days suffice. The author relates five observations in the horse and one in a man in which the cure was complete.—*Jour. de Med. Vet. et de Zoot.*

